# Supplementary material for: Making sense of the pandemic: Parent-child conversations in two cultural contexts
Source: PLoS One. 2023 Jan 23;18(1):e0280781. doi: 10.1371/journal.pone.0280781 (PMC9870112; doi:10.1371/journal.pone.0280781)
Supplement: Attachment — Submitted filename: Response to Reviewers.docx [file pone.0280781.s002.docx]

**Response letter to the reviewers**

Thank you very much for reviewing the manuscript and pointing out important caveats in the form it was first submitted. We have tried to address all of your concerns and we hope that you find the manuscript much improved. I will respond and point out the amendments point by point below.

Concerns raised by the academic editor:

1. The style of the manuscript (titles, references etc.) was aligned with the requirements of PLOS ONE.
2. The Acknowledgement section was deleted. As it was, it only doubled the information already presented in the Funding Section. The Funding Section contains all necessary information and does not need to be updated.
3. The anonymized data set is uploaded.
4. The method section is updated with information about written consent and the ethics committees that oversighted the project.

**Reviewer 1**

The introduction and especially the part about Corona experience was updated and carefully reworked in order to point out the importance of the manuscript (observational comparative study) yet be respectful of its limitations (not naturally occurring talk, but self-selected recordings). We have also taken care to clarify why emotions are expected to be mentioned.

In the method section:

- More information is provided about the preschool setting during data collection.
- It is clarified how much data was collected during the first and the second wave.
- Unfortunately, children’s language abilities were not assessed in this study. We do now refer to the need to do so in future studies in the limitation section.
- The prompt provided to parents is now presented in the method section.

In the results section:

- Poisson regression with the log link were run to analyze the data, it is now also clarified in the manuscript.
- The mean number of clauses for children and parents are also now provided in the Descriptive analyses section. In addition, proportions of the type of talk relative to the amount of talk are presented in Table 2.

Discussion:

- Unfortunately, the types of explanations for illness have not been studied in these contexts before. At the same time, we have rewritten the introduction and discussion section to provide a better foundation for hypothesis and the explanation for the results.
- The limitation of the study were expanded; both the restrictions impinged by the sample and the fact that we do not have language assessment data are highlighted.

In general:

- Inconsistences in terminology were removed.
- The manuscript was carefully proofread.

Reviewer 2

1. We rewritten the instruction (and especially “Present study” section) where we now provide more information about the slightly different focus on autonomy and relatedness and the related differences in socialization.
2. More information is provided about recruitment and the small sample size and need for more diversity is acknowledged in the limitation section of the manuscript
3. The MAXQDA program was used to manually assign codes to segments of the transcript. No automatic coding was used. This is now also clarified in the manuscript.
4. Examples were selected from among other similar ones to display the particular coding category under discussion and to illustrate the results.
5. Presentation issues were fixed, and the suggested literature added to the introduction.

We thank you again for your input and hope you find the manuscript improved!
