# Supplementary material for: Making sense of the pandemic: Parent-child conversations in two cultural contexts
Source: PLoS One. 2023 Jan 23;18(1):e0280781. doi: 10.1371/journal.pone.0280781 (PMC9870112; doi:10.1371/journal.pone.0280781)
Supplement: Attachment — Submitted filename: Response to reviewers .docx [file pone.0280781.s003.docx]

**Response to reviewers**

Thank you very much for your careful attention to the manuscript and the points that you raised! We have addressed all your concerns and hope you find the manuscript improved! Here are the changes we made in respect to reviewers’ comments:

Reviewer 1:

- As suggested by reviewer 1 Analyses were run regarding the parental education of the sample. It turned out the German mothers are more likely to have a Master’s degree or above, while Estonian mothers are more likely to have a Bachelor’s degree. A note about possible effects of this educational difference is also added to the limitations section.
- Also, analyses were run in order to establish if attending kindergarten was related to the length of the conversations and the results are reported in the manuscript.
- It is now indicated whether the utterance is provided by the child or parent in the Table. It is not possible to provide examples by parent and child at all times, as not all categories were used by both e.g. children did not use Evaluative Undefined category or parents did not use Animistic Explanations.
- The error in the heading of the second table was fixed and it is Table 2 in the revised version of the manuscript. The reference in the text is also fixed.
- Asterix were added to the values to adhere to reporting conventions.
- The mistake is fixed and an exact p value for Mann-Whitney is reported in the manuscript.
- The context where the Menendez et al study was carried out is included in the Discussion.
- The manuscript was carefully proof-read.

Reviewer 2:

- The question of gender difference in parent child conversation is an interesting question in itself. But in the current study, the difference in the number of girls and boys in the two samples is not statistically significant. Therefore, there is no reason to include gender as a control variable, especially considering the small sample size.
- We have included the information about parent age in the manuscript in order to describe the sample more. The sample demographics are now also organized in a table to provide a better overview. We originally also asked about household size, but something got lost in the translation, and we unfortunately do not have that data to provide.

Thank you very much for your valuable input and for helping us improve the quality of the manuscript!
