# Supplementary material for: Making sense of the pandemic: Parent-child conversations in two cultural contexts
Source: PLoS One. 2023 Jan 23;18(1):e0280781. doi: 10.1371/journal.pone.0280781 (PMC9870112; doi:10.1371/journal.pone.0280781)
Supplement: Attachment — Submitted filename: Response to reviewers.docx [file pone.0280781.s004.docx]

Dear Editor Sergio A. Useche,

Thank you for overseeing our manuscript! We would also like to thank the Reviewer for their suggestion. We have changed the manuscript as follows:

- We have taken into account the suggestion by Reviewer 1 and run the regression analyses with maternal education as a covariate. The results are provided in the manuscript. We also added a paragraph to the Discussion to refer to the results of these analyses.

Thank you for having our work considered!
